# Effect of acarbose and vildagliptin on plasma trimethylamine N-oxide levels in patients with type 2 diabetes mellitus: a 6-month, two-arm randomized controlled trial

**DOI:** 10.3389/fendo.2025.1575087

**Published:** 2025-05-06

**Authors:** Xinhui Yang, Xiuying Zhang, Chen Sun, Cuiling Zhao, Xiangshuang Kong, Mingming Zhao, Linong Ji, Yufeng Li

**Affiliations:** ^1^ Department of Endocrinology, Beijing Friendship Hospital Pinggu Campus, Capital Medical University, Beijing, China; ^2^ Department of Endocrinology and Metabolism, Peking University People’s Hospital, Peking University Diabetes Centre, Beijing, China; ^3^ Department of Cardiology and Institute of Vascular Medicine, Peking University Third Hospital, Beijing, China

**Keywords:** oral antidiabetic drugs, type 2 diabetes mellitus, trimethylamine N-oxide, randomized controlled trial, gut microbiota-derived metabolites

## Abstract

**Aim:**

We aimed to assess the effects of acarbose and vildagliptin on levels of plasma trimethylamine N-oxide (TMAO) and its metabolic precursor in overweight and obese patients with type 2 diabetes mellitus and ascertain the correlation between TMAO and characteristics of diabetes.

**Methods:**

This study employed a randomized, controlled, open interventional design and recruited 100 participants who were overweight/obese and newly diagnosed with type 2 diabetes at Pinggu Hospital, Beijing Friendship Hospital, Capital Medical University, between December 2016 and December 2017. Using the sealed envelope method, participants were randomly allocated (1:1) to either the acarbose group (n = 50) or the vildagliptin group (n = 50). Participants received 6 months of treatment with oral glucose-lowering medications, acarbose, or vildagliptin. Anthropometric measurements, including height, weight, waist and hip circumferences, and blood pressure, were recorded at baseline and 3 and 6 months after the intervention. Blood samples were obtained to assess blood glucose, insulin, gut hormones, TMAO, and metabolic precursors. Data analysis focused on intragroup and intergroup variations.

**Results:**

Baseline characteristics, including weight, BMI, waist and hip circumferences, blood glucose, and gut hormone levels, were comparable between the acarbose and vildagliptin groups (all *p*>0.05). Intragroup analysis indicated a significant decrease in TMAO levels at 6 months compared with baseline (adjusted *p*<0.05). L-carnitine and γ-butyrobetaine levels significantly increased at 6 months (all adjusted *p*<0.05), whereas betaine and choline levels remained non-significant throughout the intervention. Intergroup analysis revealed significantly lower TMAO levels in the acarbose group at 6 months (*p*<0.05), without significant intergroup differences in L-carnitine, γ-butyrobetaine, choline, or betaine levels (all *p*>0.05). In the acarbose group, positive correlations were observed between changes in TMAO and BMI, waist circumference, postprandial glucose, fasting insulin, fasting C-peptide, and HOMA-IR from baseline to 6 months (*p*<0.05).

**Conclusions:**

Both acarbose and vildagliptin treatments significantly reduced TMAO levels in newly diagnosed T2DM patients, with a more pronounced reduction observed in the acarbose group. Furthermore, the decline in TMAO levels correlated significantly with improvements in insulin resistance parameters.

**Clinical trial registration:**

https://clinicaltrials.gov, identifier NCT02999841.

## 1 Introduction

Diabetes mellitus represents a spectrum of metabolic disorders that are characterized by dysregulated carbohydrate metabolism, wherein diminished peripheral glucose utilization and excessive hepatic glucose production collectively drive hyperglycemia (1). As the predominant subtype accounting for 90–95% of cases, type 2 diabetes mellitus (T2DM) originates from the synergistic interplay of insulin resistance in key metabolic tissues (skeletal muscle, liver, and adipose) and progressive β-cell dysfunction. This dual pathophysiology disrupts the insulin secretion–demand equilibrium, which ultimately overwhelms compensatory mechanisms and results in relative insulin deficiency (2). In 2022, an estimated 828 million adults (those aged 18 years and older) were affected by diabetes, marking a staggering 630 million increase since 1990 (3) and imposing a global economic burden exceeding $996 billion annually (4). More than 50% of patients with diabetes in low- and middle-income countries remain undiagnosed, and this contributes to preventable complications (3, 5). Chronic hyperglycemia induces microvascular (retinopathy, nephropathy) and macrovascular complications (cardiovascular disease), accounting for the majority of the substantial global economic burden attributable to diabetes (6). Despite advances in therapeutic strategies, the intricate interplay among oral antidiabetic drugs (OADs), gut microbiota dynamics, and metabolic outcomes remains underexplored.

Emerging evidence implicates gut microbiota-derived metabolites as critical mediators of metabolic homeostasis. Among these, trimethylamine N-oxide (TMAO; (CH3)3NO), a gut microbiota-dependent metabolite derived from dietary choline and carnitine (abundant in red meat and dairy) (7, 8), has emerged as a potent biomarker and pathogenic effector in cardiometabolic diseases (9, 10). Critically, TMAO is strongly associated with T2DM development (11–15). Preclinical studies demonstrate that TMAO exacerbates IR by activating hepatic PERK/FoxO1 signaling activation, which impairs insulin receptor substrate phosphorylation (16), and by suppressing bile acid synthesis via CYP7A1 inhibition, thereby disrupting glucose homeostasis (17, 18). Zhuang et al. (14) conducted a dose-response meta-analysis encompassing 12 clinical studies with over 15,000 participants around the world and reported that circulating TMAO concentrations were positively correlated with a high risk of T2DM, with longitudinal studies corroborating its predictive value for incident T2DM in middle-aged populations (15, 19). Furthermore, patients with T2DM with elevated circulating TMAO levels are at higher risk of doubling serum creatinine, progressing to end-stage kidney disease, and mortality. TMAO is a potential biomarker for kidney function progression and mortality in patients with T2DM (20, 21).

Oral antidiabetic drugs (OADs) exhibit pleiotropic effects beyond glycemic control, particularly through gut microbiota modulation (22–24). Acarbose, an α-glucosidase inhibitor, reduces postprandial hyperglycemia by delaying carbohydrate absorption in the small intestine. Undigested carbohydrates reaching the colon undergo microbial fermentation, reshaping the gut ecosystem. In both high-starch and high-fiber diet backgrounds, acarbose treatment has been shown to increase the levels of short-chain fatty acids (SCFAs), including butyrate (25). In obese mice, acarbose enriches propionate-producing *Parasutterella*, suppressing adipose inflammation (26). Our prior clinical trials demonstrated that acarbose selectively enriches SCFA-producing bacteria in prediabetes (27) while reducing *Bacteroides* species and boosting *Bifidobacterium* members (28). Similarly, vildagliptin, a dipeptidyl peptidase-4 (DPP-4) inhibitor, prolongs incretin signaling by inhibiting glucagon-like peptide-1 (GLP-1) degradation, which may indirectly modulate microbial ecology. Evidence from a study of fecal microbiota transplantation (FMT) has indicated that the DPP-4i-altered microbiome can enhance glucose tolerance in colonized mice, concomitantly increasing the abundance of *Bacteroidetes (* 29). Our previous study suggested that both acarbose and vildagliptin monotherapy can significantly improve glycemic control and alter gut microbiota in patients with T2DM (28), however, the impact of this two medication on TMAO level remain unexplored.

This study was conducted to investigate the influence of acarbose and vildagliptin on plasma TMAO concentrations within an identical cohort of patients with T2DM and to explore the correlation between TMAO and characteristics of diabetes. Based on the role of TMAO in impairing insulin signaling and the microbiota-modulating properties of acarbose and vildagliptin, we hypothesize that both acarbose and vildagliptin reduce plasma TMAO concentrations in newly diagnosed T2DM patients, and that TMAO reduction correlates with improvements in insulin resistance (HOMA-IR).

## 2 Materials and methods

### 2.1 Participants

This study enrolled 100 adults aged 30–70 years with newly diagnosed, treatment-naïve T2DM (NDT2D) from the endocrinology outpatient clinics of Pinggu Hospital (Beijing, China) between December 2016 and December 2017. The age range was selected to encompass the peak T2DM incidence window, while excluding individuals with monogenic diabetes (age <30 years) and polypharmacy confounders among older adults (age >70 years) (30, 31).

Inclusion criteria:1) Newly diagnosed T2DM per 1999 WHO criteria (32), confirmed by a 75-g oral glucose tolerance test (OGTT), with disease duration ≤12 months and no prior glucose-lowering medication use (or ≤1 month of prior use followed by a 3-month washout). 2) Body mass index (BMI) 24.0 -30.0 kg/m², classified as overweight (24.0-27.9 kg/m²) or obese(≥28.0 kg/m²) per Chinese obesity criteria (33). 3) HbA1c 7.0–9.0% (53–75 mmol/mol), aligned with efficacy-safety thresholds for oral monotherapy in the Chinese Diabetes Guidelines (2013) (34).

Excluded criteria comprised pregnancy, lactation, or intention to conceive; gastrointestinal diseases; infectious liver diseases (hepatitis B and C); endocrine diseases affecting glucose metabolism; psychiatric disorders; alcohol dependence; neoplasia; or any other severe disease that could affect the outcomes of the study. None of the participants had consumed or injected medicines or food containing antibiotics, hormones, or probiotics for at least 1 month before the initiation of the study and had a history of weight loss, dyslipidemia, hyperglycemia, or hypertension.

All participants underwent on-site screening, including informed consent (detailing gastrointestinal risks of acarbose), medical history review, anthropometric measurements and biochemical analysis. Participants were urban residents of Pinggu District with comparable dietary and lifestyle behaviors.

### 2.2 Study design

**Figure 1:**
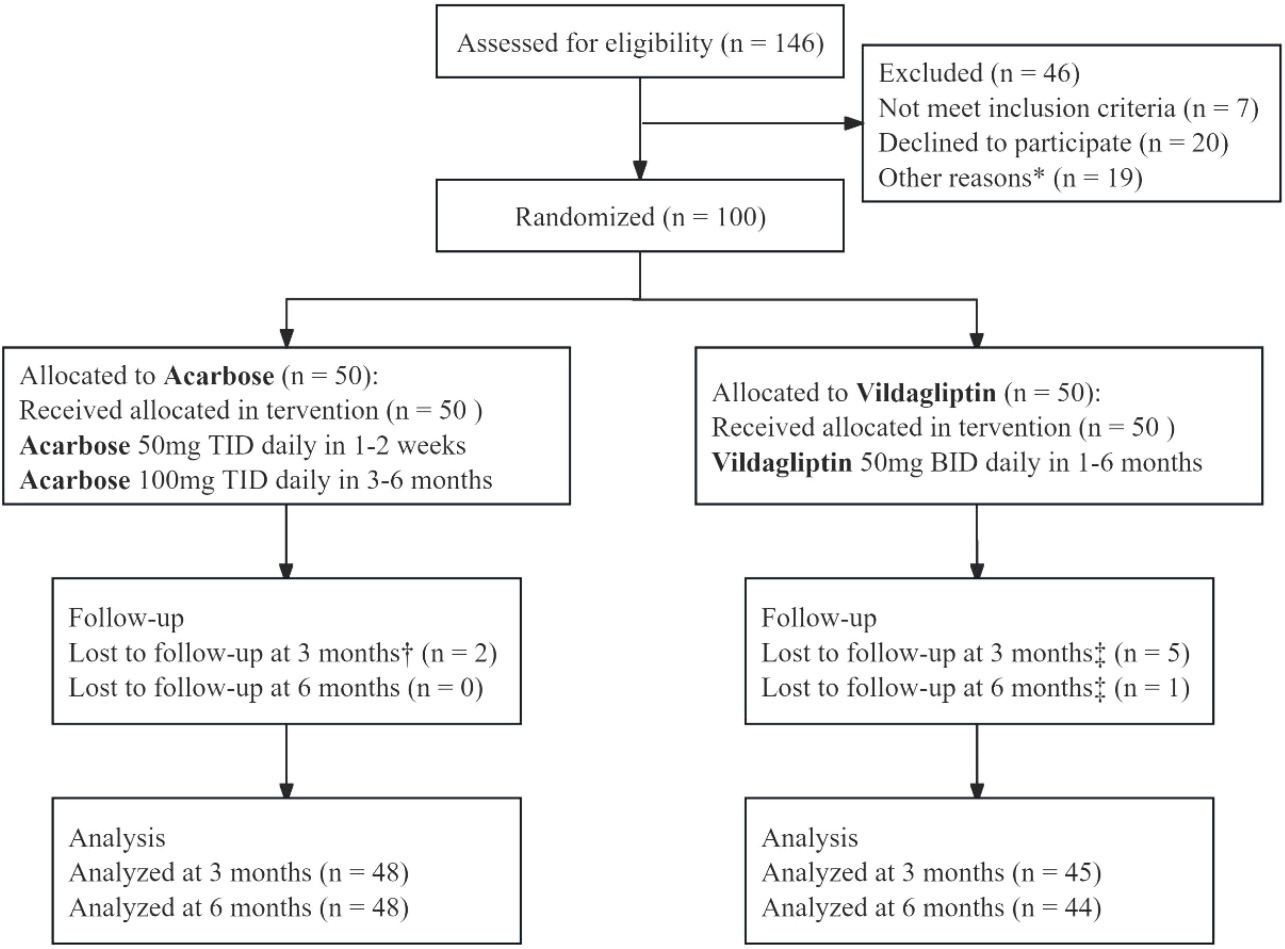
CONSORT flow diagram of a 6-month randomized controlled trial comparing vildagliptin and acarbose monotherapy in newly diagnosed type 2 diabetes mellitus. The diagram outlines participant screening (n=146), exclusion (n=46: unmet inclusion criteria [n=7], declined participation [n=20], other reasons [n=19]), randomization (n=100), and attrition. Acarbose (50 mg TID escalated to 100 mg TID at week 3) and vildagliptin (50 mg BID) were administered. Participants with 80–120% adherence were included in the per-protocol analysis (96% [48/50] acarbose, 88% [44/50] vildagliptin). *Other reasons: Participants withdrew consent (n = 12) or failed biochemical criteria (n = 7). †Acarbose group lost to follow-up: 1 relocated, 1 withdrew consent. ‡Vildagliptin group lost to follow-up: 3 lost contact, 2 withdrew consent (3 months); 1 lost contact (6 months).

This study involved biomarker exploration and data mining derived from a previously conducted exploratory, randomized, controlled, open-label intervention trial (without a placebo arm) (28) (**Figure 1**). The sample size for this exploratory trial (50 per group) was determined based on prior human gut microbial intervention studies (22–51 subjects per group) (35–37), accounting for potential attrition.

Eligible participants were randomly assigned (1:1) to the acarbose or vildagliptin group via simple randomization. A computer-generated random number sequence was created using SAS 9.0 by an independent statistician. Allocation concealment was ensured using opaque, sealed envelopes. The study treatments were as follows (1): Acarbose group (n = 50): acarbose (Bayer Medical and Health Co., Ltd., 50 mg/tablet) was initiated at 50 mg three times daily (TID) with the first meal for weeks 1-2, then escalated to 100 mg TID from week 3 onward to mitigate gastrointestinal adverse effects (e.g., bloating, flatulence). (2) Vildagliptin group (n = 50): vildagliptin (Swiss Novartis Limited, 50 mg/tablet) was administered as 50 mg twice daily (BID) before meals. All participants received telephone reminders 1–2 days prior to each visit, completing 5 telephone follow-ups and 3 on-site visits. During each visit, all participants received comprehensive lifestyle guidance according to the Chinese Diabetes Guidelines (2013) (34), which included daily caloric reduction (400–500 kcal), saturated fat restriction (<30% total fat), and ≥150 minutes/week of moderate-intensity exercise (e.g., brisk walking). Medication adherence was monitored through dosing diaries, pill counts, and returned packaging. Participants with 80–120% adherence were included in the Per Protocol Population (PPS).

### 2.3 Measurements of glucose and insulin concentrations

Blood specimens were collected via venipuncture at baseline and at 3 months (3M) and 6 months (6M) post-intervention. Glycosylated hemoglobin (HbA_1c_) was measured using cation-exchange high-pressure liquid chromatography (HPLC) method (Adams A1c HA-8160; Arkray, Kyoto, Japan). Plasma concentrations of fasting plasma glucose (FPG) and postprandial 2-h glucose (PPG) after a routine 75-g OGTT were measured using the glucose oxidase method. Fasting insulin (Fins) and postprandial 2-hour insulin (Pins) levels were determined using an electrochemiluminescence immunoassay (Elecsys 2010 system, Roche Diagnostic Ltd, Basel, Switzerland). C-peptide levels were measured using Direct Chemiluminescence (Atellica IM series; Siemens Healthcare Diagnostics).

HOMA-IR score was calculated using the following formula:


HOMA-IR=(FPG [mmol/L]×Fins [μU/mL])/22.5

HOMA-β score was calculated using the following formula:


HOMA-β=20×Fins [μU/mL]/(FPG [mmol/L]−3.5).

### 2.4 Measurement of plasma TMAO, choline, L-carnitine, betaine, and γ-butyrobetaine levels

Levels of TMAO, choline, L-carnitine, betaine and γ-butyrobetaine were quantified using stable isotope dilution liquid chromatography–tandem mass spectrometry, as described previously (38). Briefly, 20 μL plasma were aliquoted into a 1.5-mL tube and mixed with 80 μL 10 μM internal standard composed of d9-TMAO, d9-choline, d9-carnitine, d11-betaine, and d9-γ-butyrobetaine in methanol. Protein in the samples was precipitated and the supernatant was recovered following centrifugation at 20,000 g at 4°C for 10 min, and the resulting supernatants were analyzed by injection onto a silica column (2.0*150 mm, Luna 5u Silica 100A; Cat. No. 00F-4274-B0, Phenomenex, Torrance, CA) at a flow rate of 0.4 mL/min using a LC-20AD Shimadazu pump system, SIL-20AXR autosample interfaced with an API 5500Q-TRAP mass spectrometer (AB SCIEX, Framingham, MA). A discontinuous gradient was employed to separate the analytes by mixing solvent A (0.1% propanoic acid in water) with solvent B (0.1% acetic acid in methanol) at different ratios, starting from 2% B linearly to 95% B, over 5.0 min, then held for 1.0 min, and then back to 2% B. Analytes were monitored using electrospray ionization in positive-ion mode with multiple reaction monitoring (MRM) of precursor and characteristic product-ion transitions of TMAO at m/z 76→58, d9-TMAO at m/z 85 →66, choline at m/z 104 →59.8, d9-choline at m/z 113.2→68.9, carnitine at m/z 162.1→103, d9-carnitine at m/z 171.1→102.8, betaine at m/z 118 →59, d11-betaine at m/z 129.1→65.9, γ-butyrobetaine at m/z 146.1→87, d9-γ-butyrobetaine at m/z 155.1→87, respectively.

### 2.5 Other measurements

The fasting serum lipid profile, including total cholesterol (TC), triglyceride (TG), high-density lipoprotein cholesterol (HDL-C), and low-density lipoprotein cholesterol (LDL-C) levels, was ascertained using an automated biochemical analyzer. Serum concentrations of gut hormones, including active glucagon-like peptide-1 (GLP-1), ghrelin, peptide YY (PYY), and leptin, were quantified in the fasting state using standard enzyme-linked immunosorbent assay (ELISA) kits (EGLP-35K, EZGRT-89K, EZHPYYT66K, Merck Millipore, USA). The radioimmunoassay method (RK-069-04, Phoenix Peptide, USA) was used to measure serum cholecystokinin (CCK). Each participant was subjected to a series of anthropometric assessments, including body weight, height, waist and hip circumference, and systolic and diastolic blood pressure (SBP and DBP, respectively). BMI was calculated as the weight divided by height squared (kg/m^2^). The waist-to-hip ratio (WHR) was calculated as waist circumference divided by hip circumference. All anthropometric measurements were taken by registered nurses at the baseline, 3M, and 6M visits.

### 2.6 Statistical analysis

We analyzed the differences between the vildagliptin and acarbose treatment arms in clinical variables at baseline. Shapiro–Wilk test for determining whether continuous variables are normally distributed. Normally distributed quantitative variables are described as mean and standard deviation (SD); the independent samples *t*-test was used for intergroup comparisons. Non-normally distributed quantitative variables are presented as median (25^th^, 75^th^ percentiles), and the Mann–Whitney *U* test was used to compare the two groups. Longitudinal changes in gut microbial metabolites (TMAO, L-carnitine, betaine, choline, and γ-butyrobetaine) across three timepoints (baseline, 3 months [3M], and 6 months [6M]) were evaluated using the Friedman test, a non-parametric repeated-measures analysis. *Post hoc* pairwise comparisons were conducted with Dunn’s test, adjusted for multiple testing via Bonferroni correction. To compare the degrees of posttreatment changes in clinical variables of different treatment group, we normalized the changes (Δ) of each variable from the baseline examination to 6M of the drug intervention. For each individual, change from baseline of a given variable was calculated using the following equation: . where Base_variable_ and M6_variable_ are the values of a given variable from the same individual at baseline and 6M in the acarbose and vildagliptin groups, respectively. To investigate the correlation between the levels of gut microbial metabolites and glucose metabolic variables during treatment with different agents, Spearman bivariate correlation analysis was conducted between the changing glucose metabolic characteristics and plasma levels of TMAO, L-carnitine, betaine, choline, and γ-butyrobetaine at 6M. All analyses were two-tailed, with *p* < 0.05 considered statistically significant, and performed in SPSS 25.0 and GraphPad Prism 9.5.0. A correlation heatmap was drawn using ChiPlot (https://www.chiplot.online/) (accessed 10 October 2024).

### 2.7 Ethics approval

This study was approved by the Ethics Committee of the Peking University Health Science Center (2015PHB175-01; Date: 18 November 2015) and was conducted in compliance with the Declaration of Helsinki. The study was registered at ClinicalTrials.gov (NCT02999841). All participants provided written informed consent.

## 3 Results

### 3.1 Baseline characteristics of participants

One hundred eligible patients with NDT2D (54 men and 46 women) were randomly assigned (1:1) to either the acarbose or vildagliptin treatment group. Seven participants were lost to follow-up at the 3-month visit and eight at the 6-month visit after starting the intervention. In total, 92 participants completed the 6-month trial: 48 in the acarbose group and 44 in the vildagliptin group. No serious drug-related adverse events were observed.

Baseline clinicodemographic characteristics were comparable between the study groups (**Table 1**). No significant differences were observed in age (*p*= 0.434) or sex distribution (*p*= 0.108). Glycemic parameters (HbA_1c_, FPG, PPG, Fins, Pins, HOMA-β, HOMA-IR), obesity measures (body weight, BMI, waist circumference, and hip circumference), and lipid profiles (TC, TG, LDL-C) were balanced between the two groups (*p*>0.05 for all; **Table 1**). However, HDL-C levels were significantly higher in the vildagliptin group (*p*=0.030). Additionally, there were no significant intergroup differences in blood pressure, intestinal hormone levels, or adipokine levels (*p*>0.05 for all; **Table 1**). More detailed information has been published (28).

**Table 1:** Demographic and metabolic characteristics of the participants.

Characteristics	Acar (n = 50)	Vild (n = 50)	*P*
Demographic characteristics			
Male/female, n	31/19	23/27	0.108
Age, years	51.90 (9.75)	50.44 (8.82)	0.434
Glycaemic characteristics			
HbA_1c_, mmol/mol	60.11 (56.28, 67.21)	59.02 (54.10, 65.36)	0.377
HbA_1c_, %	7.65 (7.30, 8.30)	7.55 (7.10, 8.13)	0.377
FPG, mmol/l	8.32 (7.48, 9.61)	8.75 (7.67, 9.40)	0.903
PPG, mmol/l	12.20 (11.18, 13.91)	13.13 (11.01, 15.06)	0.315
Fins, pmol/l	10.65 (6.36, 14.34)	9.96 (7.07, 12.42)	0.617
Pins, pmol/l	32.57 (21.17, 45.69)	31.59 (24.22, 50.45)	0.760
HOMA-IR	4.11 (2.44, 5.56)	3.90 (2.74, 5.20)	0.845
HOMA-β	39.71 (26.56, 54.63)	38.35 (23.71, 51.87)	0.663
Fc-peptide	1.86 (1.54, 2.32)	1.67 (1.40, 2.24)	0.369
Pc-peptide	4.22 (3.40, 5.53)	4.60 (3.93, 5.19)	0.293
Obesity variables			
Weight, kg	72.71 (8.50)	70.85 (9.66)	0.309
BMI, kg/m²	26.83 (1.81)	26.87 (1.81)	0.923
Waist, cm	94.18 (6.57)	92.71 (6.63)	0.268
Hip, cm	98.77 (5.40)	98.16 (5.38)	0.573
WHR	0.95 (0.05)	0.94 (0.05)	0.333
Blood lipids			
TC, mmol/l	5.04 (4.63, 5.96)	5.12 (4.70, 5.77)	0.763
TG, mmol/l	1.99 (1.30, 3.74)	1.97 (1.24, 2.69)	0.559
HDL-C, mmol/l	1.20 (0.20)	1.29 (0.21)	0.030
LDL-C, mmol/l	2.98 (0.78)	2.93 (0.64)	0.756
Blood pressure			
mSBP, mmHg	129.01 (14.47)	130.74 (14.84)	0.556
mDBP, mmHg	78.39 (8.91)	77.33 (10.00)	0.577
Gut hormones and adipokines			
Leptin, ng/ml	11.12 (7.05, 17.47)	17.57 (6.19, 24.03)	0.138
GLP-1, pmol/l	3.14 (2.90, 3.38)	3.14 (2.99, 3.68)	0.304
CCK, pg/ml	13.78 (7.36, 23.21)	9.51 (5.26, 19.65)	0.224
Ghrelin, pg/ml	368.04 (253.23, 608.82)	411.38 (301.63, 546.94)	0.780
PYY, pg/ml	117.39 (92.26, 174.99)	133.11 (107.62, 152.23)	0.506

Continuous data are presented as mean (SD) or median (25^th^, 75^th^ percentiles). *P*-values obtained from two independent-samples *t*-test and Mann–Whitney *U* test (continuous data) or the χ^2^ test (categorical data).

Acar, acarbose treatment group; Vild, vildagliptin; TC, total cholesterol; TG, triglyceride; mDBP, mean DBP; mSBP, mean SBP.

### 3.2 Gut microbiota metabolite dynamics during treatment

Longitudinal analysis of plasma gut microbiota metabolites revealed no baseline intergroup differences in TMAO, L-carnitine, betaine, choline, or γ-butyrobetaine concentrations (*p* > 0.05 for all; **Table 2**).At 6 months, TMAO concentrations were significantly lower in the acarbose group versus vildagliptin (*p* < 0.05), while other metabolites showed no intergroup divergence (*p*>0.05 for all; **Table 2**).

**Table 2:** Intergroup differences in the levels of gut microbial metabolites.

Characteristics	Baseline			3 Months			6 Months		
	Acar (n = 50)	Vild (n = 50)	*P*	Acar (n = 48)	Vild (n = 45)	*P*	Acar (n = 48)	Vild (n = 44)	*P*
TMAO	3.84 (2.24, 5.22)	3.35 (2.14, 5.45)	0.814	2.06 (0.72,9.29)	2.35 (1.62, 5.63)	0.829	1.03 (0.31, 2.67)	1.74 (1.22, 2.52)	0.013
L-Carnitine	45.90 (38.80, 54.50)	49.40 (42.40, 59.23)	0.050	52.05 (44.48, 61.43)	55.20 (44.03, 63.20)	0.636	54.80 (47.93, 64.70)	58.35 (49.18, 67.93)	0.329
Betaine	38.80 (32.30, 49.30)	40.60 (32.53, 49.25)	0.732	41.30 (30.88, 51.63)	37.40 (30.70, 49.80)	0.475	37.90 (33.55, 48.80)	40.95 (32.88, 50.18)	0.543
Choline	6.20 (4.78, 7.27)	6.08 (4.96, 7.39)	0.924	5.98 (4.90, 7.95)	6.34 (5.54, 7.74)	0.315	6.33 (4.93, 7.34)	6.46 (5.68, 7.83)	0.118
γ-Butyrobetaine	0.61 (0.53, 0.78)	0.72 (0.54, 0.84)	0.110	0.70 (0.61, 0.94)	0.74 (0.59, 0.98)	0.713	0.75 (0.67, 0.93)	0.76 (0.68, 0.91)	0.836

Continuous data are presented as the median (25th, 75th percentiles). *P*-values obtained from the Mann–Whitney U test.

**Figure 2:**
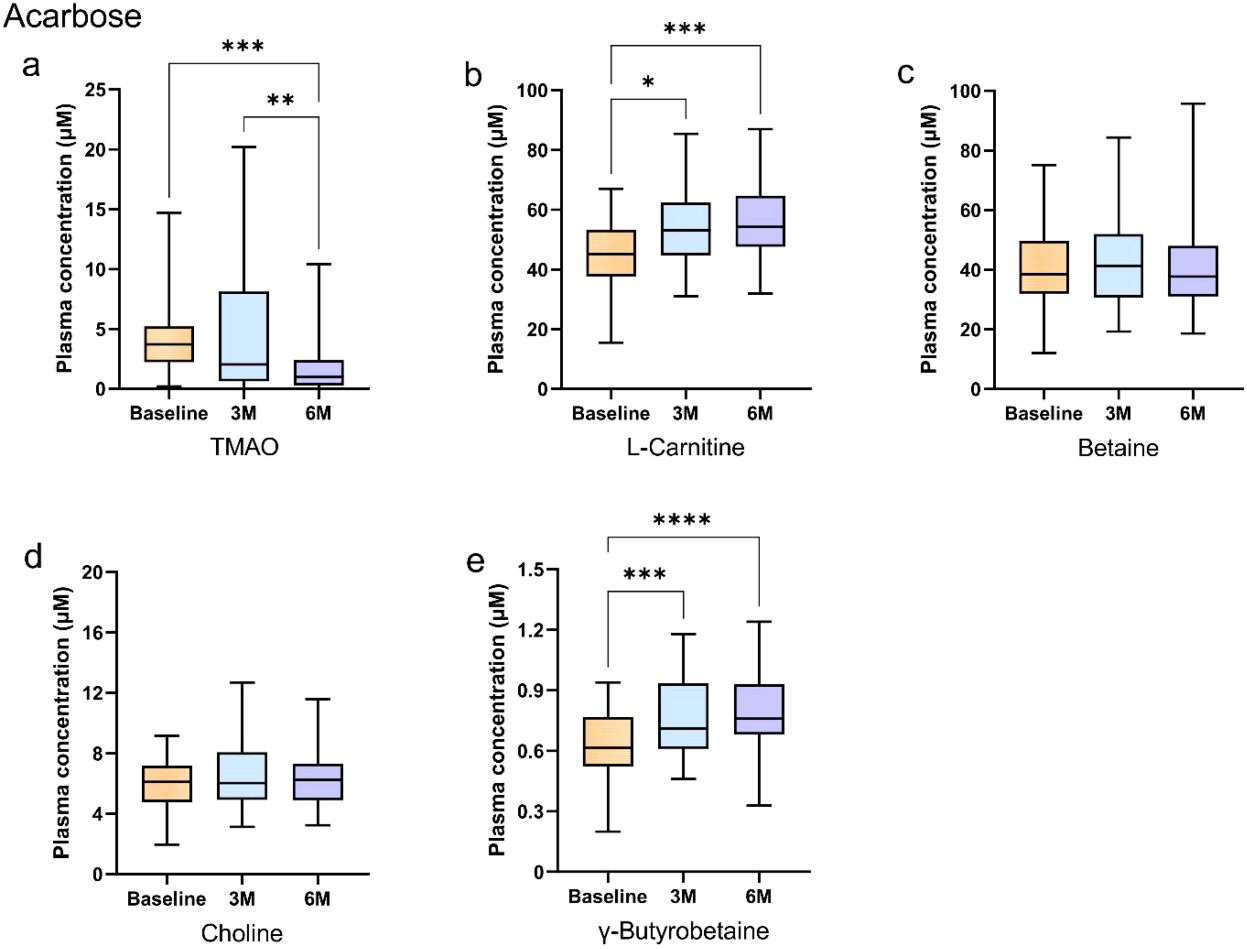
Longitudinal changes in plasma gut microbiota metabolites during acarbose treatment in newly diagnosed type 2 diabetes patients. **(A–E)** Box plots show longitudinal changes in plasma concentrations of **(a)** trimethylamine N-oxide (TMAO), **(b)** L-carnitine, **(c)** betaine, **(d)** choline, and **(e)** γ-butyrobetaine at baseline (orange), 3 months (blue), and 6 months (purple). Statistical analysis was performed using the Friedman test (non-parametric repeated measures) followed by Dunn’s *post hoc* test with Bonferroni correction for pairwise comparisons. Significance levels: *Bonferroni-adjusted *p*<0.05; **adjusted *p*<0.01; ***adjusted *p*<0.001; ****adjusted *p*<0.0001. Data are presented as median (25th, 75th). Detailed data are presented in **Supplementary Tables 1, 2**.

Intragroup comparisons using the Friedman test demonstrated time-dependent changes. In the acarbose group, plasma TMAO concentrations decreased significantly at 6 months compared to baseline and 3 months (Bonferroni-adjusted *p* < 0.05; **Figure 2a**). L-carnitine and γ-butyrobetaine concentrations increased at 3 and 6 months versus baseline (Bonferroni-adjusted *p* < 0.05; **Figures 2b, e**), while betaine and choline remained stable (Bonferroni-adjusted *p* > 0.05; **Figures 2c, d**).

**Figure 3:**
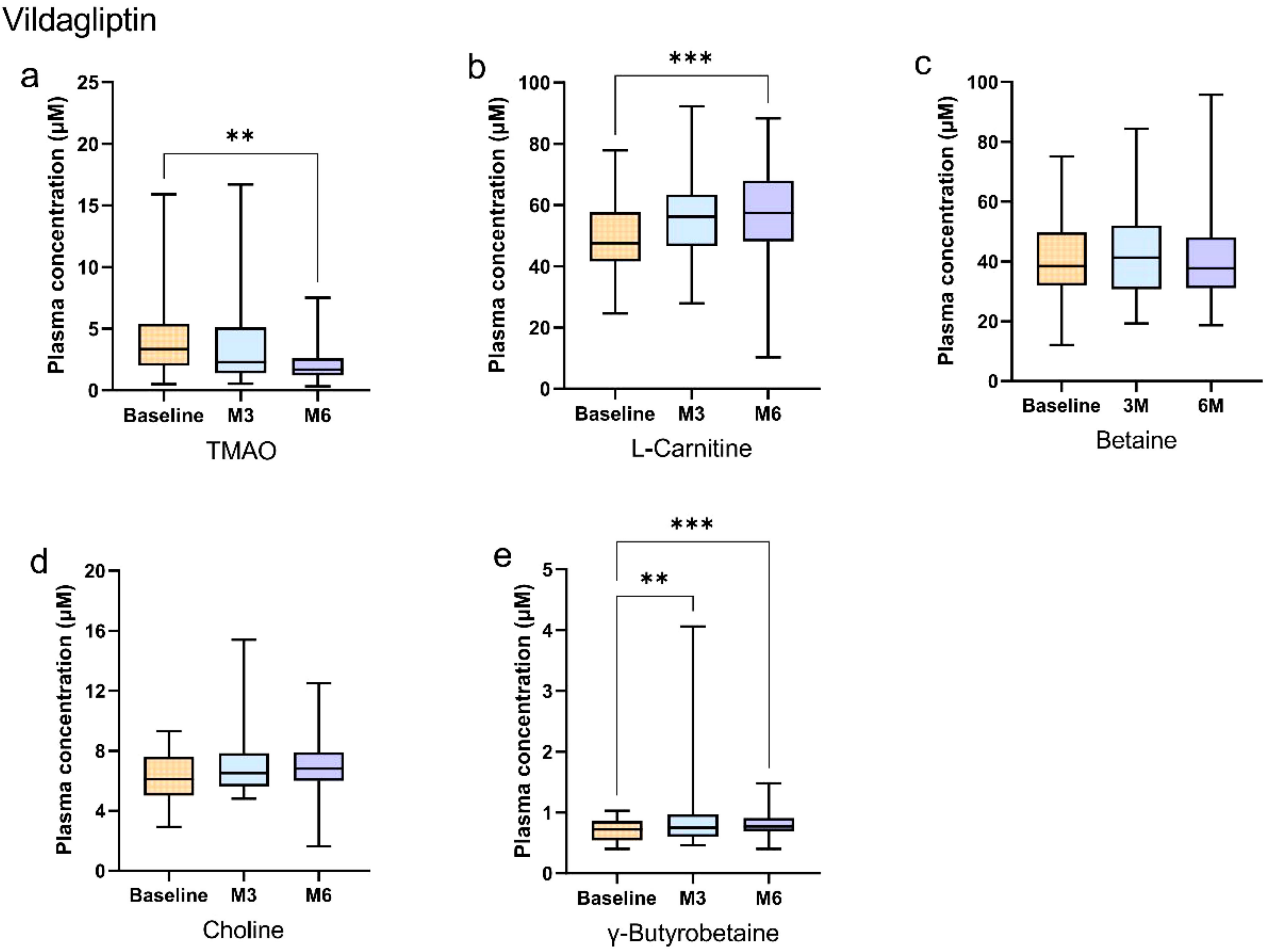
Longitudinal changes in plasma gut microbiota metabolites during vildagliptin treatment in newly diagnosed type 2 diabetes patients. **(a–e)** Box plots show longitudinal changes in plasma concentrations of **(a)** trimethylamine N-oxide (TMAO), **(b)** L-carnitine, **(c)** betaine, **(d)** choline, and **(e)** γ-butyrobetaine at baseline (orange), 3 months (blue), and 6 months (purple). Statistical analysis was performed using the Friedman test (non-parametric repeated measures) followed by Dunn’s *post hoc* test with Bonferroni correction for pairwise comparisons. Significance levels: *Bonferroni-adjusted *p*<0.05; **adjusted *p*<0.01; ***adjusted *p*<0.001; ****adjusted *p*<0.0001. Data are presented as median (25th, 75th). Detailed data are presented in **Supplementary Tables 1, 2**.

In the vildagliptin group, plasma TMAO concentrations decreased significantly at 6 months compared to baseline (Bonferroni-adjusted *p* < 0.05; **Figure 3a**). L-carnitine increased at 6 months versus baseline (Bonferroni-adjusted *p* < 0.05; **Figure 3b**), while γ-butyrobetaine concentrations increased progressively at both 3 and 6 months (Bonferroni-adjusted *p* < 0.05; **Figure 3e**). In contrast, betaine and choline concentrations remained stable (*p* > 0.05; **Figures 3c, d**). The details of the statistical analysis are available in the **Supplementary Material**.

### 3.3 Association of changes in gut microbiota metabolites with glucose metabolic characteristics

In our prior study, both acarbose and vildagliptin demonstrated significant improvements in glycemic control, lipid profiles, and blood pressure in newly diagnosed type 2 diabetes patients after 6 months of intervention (28). Building on these findings, we investigated correlations between gut microbial metabolites and glucose metabolic variables in the current work.

**Figure 4:**
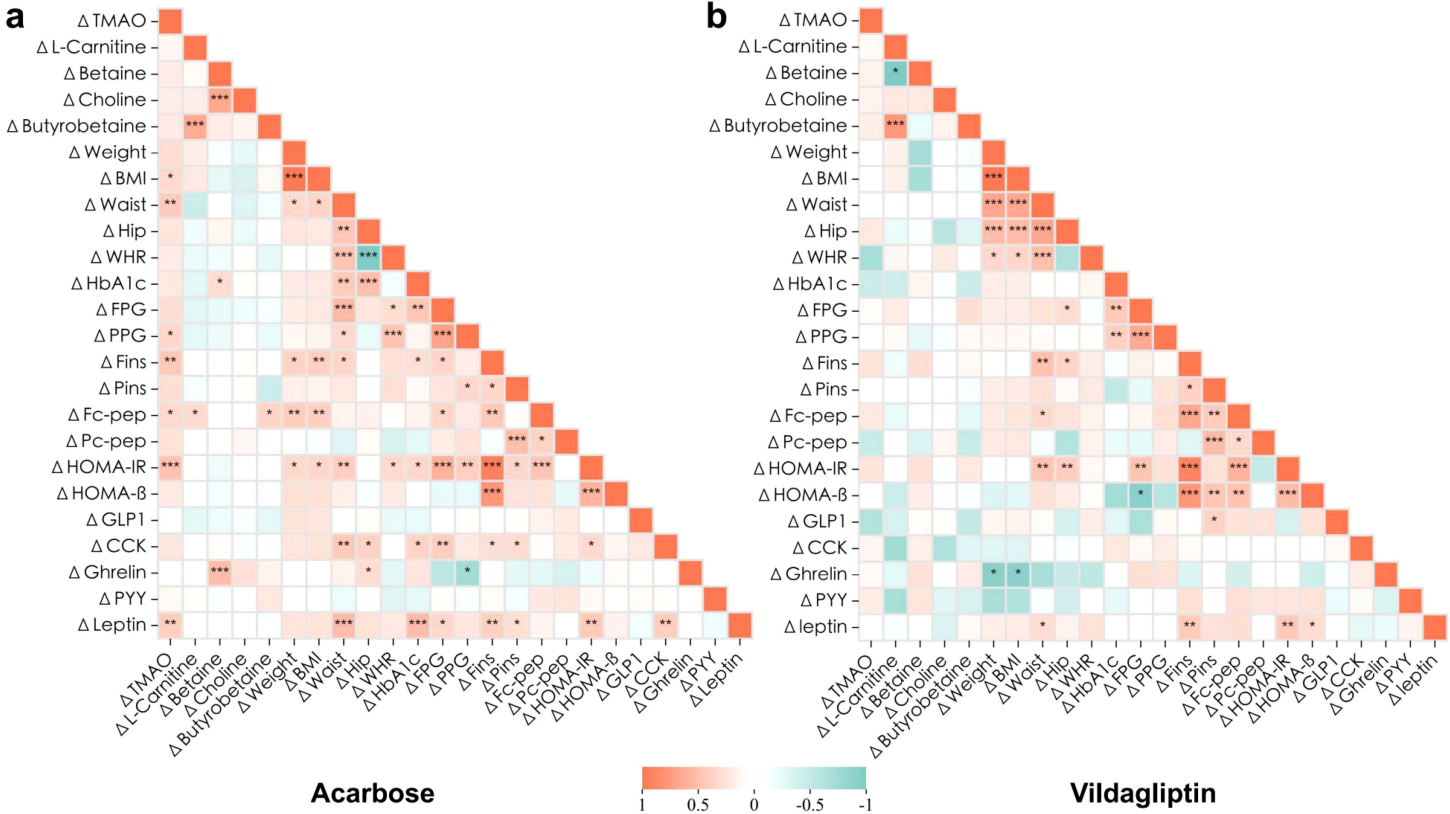
Association of changes in the clinical metabolic characteristics and plasma levels of gut microbiota metabolites after 6-month intervention. Heatmaps resulting from Spearman rank correlation coefficients (Spearman’s rho) of the changes () of each variable to its baseline with consistent responses to 6-month acarbose **(a)** or vildagliptin **(b)** treatment. *p<0.05, **p<0.01, ***p<0.001. Red and green indicate that the two variables are positively and negatively correlated, respectively. Detailed statistical data are presented in **Supplementary Table 3**.

Spearman bivariate correlation analysis at 6 months demonstrated significant associations in the acarbose group. ΔTMAO correlated positively with changes in BMI (*r* = 0.323), waist circumference (*r* = 0.423), PPG (*r* = 0.338), Fins (*r* = 0.436), fasting C-peptide (*r* = 0.328), HOMA-IR (*r* = 0.484), and leptin (*r* = 0.396) (*p* < 0.05). ΔBetaine correlated with Δghrelin (*r* = 0.524, *p* < 0.001), and both ΔL-carnitine and Δγ-butyrobetaine correlated with Δfasting C-peptide (*r* = 0.319, *p* = 0.037; *r* = 0.360, *p* = 0.017; **Figure 4**, **Supplementary Table 3**).

By contrast, no significant correlations were observed between changes in gut microbiota metabolites and metabolic parameters in the vildagliptin group *(p* > 0.05). Notably, a strong positive correlation between ΔL-carnitine and Δγ-butyrobetaine was consistent across both groups (*r* = 0.644, *p* < 0.001; *r* = 0.771 *p* < 0.001; **Figure 4**, **Supplementary Table 3**), indicating coordinated regulation of these metabolites.

## 4 Discussion

Our RCT provides novel evidence that both acarbose and vildagliptin, two widely used oral antidiabetic agents, significantly reduce plasma TMAO levels in patients with newly diagnosed T2DM. These findings extend the previous work on gut microbiota remodeling by these agents (28) and align with emerging insights into microbial contributions toward diabetes-induced dysmetabolism (19). Specifically, both drugs attenuated the carnitine→γ-butyrobetaine→TMA metabolic axis, which implicates a shared downstream effect on TMAO synthesis despite distinct pharmacological mechanisms.

### 4.1 Gut microbiota-driven TMAO biosynthesis

The gut microbiota plays a central role in TMAO biosynthesis. Bioinformatics suggested that gut microbiota can convert carnitine, betaine, and choline to TMA, through the utilization of three TMA-lyase complexes, namely cntA/B, yeaW/X, and cutC/D (39). Choline, abundant in egg yolk and red meat (40), is predominantly metabolized by *Lachnoclostridium* and *Intestinibacter* (*Firmicutes*) via cutC/D, genera enriched in cardiometabolic disorders (39, 41). Active L-carnitine is mainly found in meat and dairy products (42). For L-carnitine catabolism, *Proteobacteria* (e.g., *Escherichia* and *Shigella*) directly cleave its C-N bond via cntA/B-encoded oxygenases in the cecum (43, 44), while *Emergencia timonensis* (*Actinobacteria*) employs an alternative pathway involving γ-butyrobetaine as a transient intermediate through uncharacterized oxidoreductases (45–47). Owing to the presence of the “enterohepatic axis”, TMA can be rapidly oxidized to TMAO by hepatic FMO3 and subsequently released into the circulation (8). In the present study, the observed reduction in TMAO and accumulation of L-carnitine and γ-butyrobetaine suggest that both acarbose and vildagliptin impede the metabolic pathway of carnitine → γ-butyrobetaine → TMA, albeit through distinct mechanisms. To elucidate the impact of these two drugs on the TMAO metabolic pathway, it is important to consider the complex interplay between pharmacological interventions and the gut microbiome.

### 4.2 Pharmacological modulation of the TMAO pathway

As a first-line α-glucosidase inhibitor (AGI), acarbose delays small intestinal carbohydrate absorption, thereby reducing postprandial hyperglycemia. Undigested carbohydrates reaching the colon undergo microbial fermentation, which reshapes the gut ecosystem (48, 49). This microbiota-dependent effect may underlie its therapeutic benefits in T2DM. In mice fed with either a high-starch or high-fiber diet, acarbose increased *Bacteroidaceae* and *Bifidobacteriaceae* while reducing *Akkermansia muciniphila* and *Bacteroidales S24-7*, accompanied by elevated SCFAs (25, 50). In humans, however, our prior work revealed host-specific divergence: 6-month acarbose treatment decreased gut microbial alpha diversity and *Bacteroides* species, but enriched *Streptococcus* (*S. sanguinis, S. salivarius)* and *Bifidobacterium (* 28).

Vildagliptin, a DPP-4 inhibitor, enhances endogenous GLP-1 activity to improve glycemic control (51). While its glucose-lowering effects are partially microbiota-mediated, the specific taxonomic shifts differ across studies. In animal studies, sitagliptin increased *Bacteroidetes* abundance in mice fed a high-fat diet, correlating with improved glucose homeostasis (29). In contrast, our previous study yielded differing results: vildagliptin enriched *Bifidobacterium adolescentis* and *Intestinibacter bartlettii*, while reducing *Paraprevotella* spp., *Parabacteroides distasonis* and *Bacteroides* spp.(e.g. *B. finegoldii, B. caccae*) *(* 28). Notably, a cross-sectional analysis observed a heightened abundance of these two *Bacteroides* species in treatment-naïve patients with T2DM as opposed to those with prediabetes or normal glucose tolerance (52), underscoring their role in diabetic dysbiosis.

Although these two drugs differ in their gastrointestinal mechanisms of glycemic control, our study indicates that they elicit comparable effects on the gut microbiota, particularly with respect to the proliferation of *B. adolescentis* and the reduction of several species belonging to the phylum *Bacteroidetes* (*B. caccae* and *B. finegoldii*). This overlap may explain their similar metabolic outcomes. Acarbose’s stronger TMAO-lowering effect could stem from its direct impact on microbial carbohydrate fermentation, which selectively depletes TMA-lyase-producing taxa. Conversely, vildagliptin’s milder effect might involve indirect GLP-1-mediated modulation of gut ecology. Crucially, baseline TMAO levels were comparable between groups, ruling out preexisting differences and emphasizing drug-induced microbial reprogramming.

### 4.3 Associations between TMAO and insulin resistance

After 6 months of treatment with acarbose, the blood glucose levels of participants were effectively controlled, including decreases in FPG, PPG, HbA_1c_ and HOME-IR, with significant decreases in body weight and BMI (28). IR is defined as a state of decreased insulin sensitivity and/or responsiveness. This study, we used HOMA-IR to quantify the extent of IR in an individual and calculated it by combining the FPG and fin levels. A reduction in the HOMA-IR is typically indicative of enhanced insulin sensitivity. In the present study, we found a positive correlation between the changes in TMAO and PPG, Fin, fasting C-peptide, and HOMA-IR after 6-month acarbose treatment, suggesting a mechanistic interplay between gut microbiota-derived TMAO and IR.

Experimental studies corroborate these clinical observations. Dietary TMAO supplementation exacerbates impaired glucose tolerance in high-fat diet-fed mice by disrupting hepatic insulin signaling and inducing adipose tissue inflammation, thereby elevating HOMA-IR indices (53). FMO3 is an enzyme expressed primarily in the liver and catalyzes the conversion of TMA to TMAO (8). *In vitro* studies have confirmed that FMO3 is repressed by insulin; however, its expression is increased in obese and insulin-resistant male mice, mirroring a similar increase observed in humans. Notably, the experimental suppression of FMO3 in insulin-resistant mice effectively inhibited FoxO1, a key metabolic regulatory node, and completely circumvented the progression of hyperglycemia, hyperlipidemia, and atherosclerosis (54). In parallel with these findings, another mouse study revealed a sophisticated mechanism through which TMAO exacerbates metabolic dysfunction. This study indicates that TMAO binds to and activates the endoplasmic reticulum stress kinase PERK (EIF2AK3), thereby triggering the transcription factor FoxO1 in a PERK-dependent cascade. This signaling pathway culminates in metabolic impairment. Interventions targeting TMAO reduction, whether via microbiota modulation or FMO3 inhibition, downregulate PERK-FoxO1 signaling, thereby ameliorating metabolic dysfunction (16). These findings collectively implicate TMAO as a mediator of IR through multiple pathways, yet key questions remain unresolved. Whether TMAO reduction directly enhances insulin sensitivity or merely reflects acarbose-induced microbiota remodeling requires causal validation through fecal microbiota transplantation studies.

Thus, based on the results of the present study, we clarify that both acarbose and vildagliptin treatments significantly reduced TMAO levels in newly diagnosed T2DM patients, with a more pronounced reduction observed in the acarbose group. Furthermore, the decline in TMAO levels demonstrated a significant association with improvements in insulin resistance parameters.

Our study also has several strengths. Primarily, this two-arm RCT employed stringent inclusion criteria and meticulous follow-up protocols, ensuring high-quality data collection. Additionally, we comprehensively investigated TMAO dynamics by analyzing not only TMAO itself but also its four key precursors (L-carnitine, betaine, choline, and γ-butyrobetaine), an approach rarely adopted in prior studies. Third, by leveraging longitudinal blood sampling, this work represents the first exploration of acarbose and vildagliptin effects on TMAO metabolism in a Chinese T2DM population, enhancing the translational relevance of our findings.

Despite these advances, there are some limitations. First, as an exploratory RCT designed to investigate gut microbiota-mediated mechanisms, the sample size (n = 50/group) was determined based on prior studies, rather than a formal power analysis. While this design aligns with the exploratory nature of our hypothesis-generating objectives, future trials with larger cohorts are needed to validate these findings and assess subgroup-specific effects. Second, although our 6-month data demonstrate progressive TMAO reduction, the study duration may not represent the optimal therapeutic window. The long-term efficacy of glucose-lowering drugs on TMAO dynamics—including whether suppression stabilizes or continues to decline—remains unknown. Future trials should extend follow-up to 9–12 months to clarify the durability of TMAO reduction and its relationship to glycemic control and cardiovascular outcomes. Third, while microbial shifts correlated with TMAO reduction, our analysis did not pinpoint specific taxa responsible for TMAO metabolism (e.g., cntA/B- or cutC/D-harboring bacteria) or establish causality. Mechanistic validation through animal studies, fecal microbiota transplantation (FMT), or targeted metagenomic sequencing of TMA-lyase genes is essential to confirm these associations.

## Data Availability

The raw data supporting the conclusions of this article will be made available by the authors, without undue reservation.
