# Supplementary material for: Effect of acarbose and vildagliptin on plasma trimethylamine N-oxide levels in patients with type 2 diabetes mellitus: a 6-month, two-arm randomized controlled trial
Source: Front Endocrinol (Lausanne). 2025 May 6;16:1575087. doi: 10.3389/fendo.2025.1575087 (PMC12088947; doi:10.3389/fendo.2025.1575087)
Supplement: Supplementary file 1 [file Table1.docx]

Supplementary Material

**Supplementary Table 1 Longitudinal changes in plasma gut microbiota metabolites during acarbose and vildagliptin treatment in newly diagnosed type 2 diabetes patients.**

| Variables | Baseline | 3 Months | 6 Months | Wald *χ*² | *p* |
| --- | --- | --- | --- | --- | --- |
| Acarbose Group |  |  |  |  |  |
| TMAO | 3.84 (2.24, 5.22) | 2.06 (0.72, 9.29) | 1.03 (0.31, 2.67) ab | 15.674 | <0.001 |
| L-Carnitine | 45.90 (38.80, 54.50) | 52.05 (44.48, 61.43) a | 54.80 (47.93, 64.70) a | 16.326 | <0.001 |
| Betaine | 38.80 (32.30, 49.30) | 41.30 (30.88, 51.63) | 37.90 (33.55, 48.80) | 1.163 | 0.559 |
| Choline | 6.20 (4.78, 7.27) | 5.98 (4.90, 7.95) | 6.33 (4.93, 7.34) | 2.468 | 0.291 |
| γ-Butyrobetaine | 0.61 (0.53, 0.78) | 0.70 (0.61, 0.94) a | 0.75 (0.67, 0.93) a | 31.349 | <0.001 |
| Vildagliptin Group |  |  |  |  |  |
| TMAO | 3.35 (2.14, 5.45) | 2.35 (1.62, 5.63) | 1.74 (1.22, 2.52) a | 10.167 | 0.006 |
| L-Carnitine | 49.40 (42.40, 59.23) | 55.20 (44.03, 63.20) | 58.35 (49.18, 67.93) a | 15.744 | <0.001 |
| Betaine | 40.60 (32.53, 49.25) | 37.40 (30.70, 49.80) | 40.95 (32.88, 50.18) | 0.158 | 0.924 |
| Choline | 6.08 (4.96, 7.39) | 6.34 (5.54, 7.74) | 6.46 (5.68, 7.83) | 3.600 | 0.165 |
| γ-Butyrobetaine | 0.72 (0.54, 0.84) | 0.74 (0.59, 0.98) a | 0.76 (0.68, 0.91) a | 15.928 | <0.001 |

Friedman test was used for repeated measures data and Dunn's test was used for multiple comparisons (paired comparisons), respectively. a: levels in this group statistically differed from those at baseline, Bonferroni-adjusted *p*<0.05. b: levels in this group statistically differed from those at 3 months, Bonferroni-adjusted *p*<0.05.
