# Supplementary material for: Effect of acarbose and vildagliptin on plasma trimethylamine N-oxide levels in patients with type 2 diabetes mellitus: a 6-month, two-arm randomized controlled trial
Source: Front Endocrinol (Lausanne). 2025 May 6;16:1575087. doi: 10.3389/fendo.2025.1575087 (PMC12088947; doi:10.3389/fendo.2025.1575087)
Supplement: Supplementary file 2 [file Table2.docx]

Supplementary Material

**Supplementary Table 2 Paired comparisons of intragroup differences at prespecified timepoints in the levels of gut microbiota metabolites in NDT2D patients treated with acarbose or vildagliptin**

| Comparative variables | | Test statistic | SE | Normalisation test statistic | *p* | Adjusted-*p* |
| --- | --- | --- | --- | --- | --- | --- |
| Acarbose group | |  |  |  |  |  |
| TMAO | Baseline vs. 3M | 0.116 | 0.216 | 0.539 | 0.590 | 1.000 |
|  | Baseline vs. 6M | 0.791 | 0.216 | 3.666 | <0.001 | 0.001 |
|  | 3M vs. 6M | 0.674 | 0.216 | 3.127 | 0.002 | 0.005 |
| L-Carnitine | Baseline vs. 3M | -0.628 | 0.216 | -2.911 | 0.004 | 0.011 |
|  | Baseline vs. 6M | -0.837 | 0.216 | -3.882 | <0.001 | <0.001 |
|  | 3M vs. 6M | -0.209 | 0.216 | -0.970 | 0.332 | 0.995 |
| γ-Butyrobetaine | Baseline vs. 3M | -0.773 | 0.213 | -3.624 | <0.001 | <0.001 |
|  | Baseline vs. 6M | -1.170 | 0.213 | -5.490 | <0.001 | <0.001 |
|  | 3M vs. 6M | -0.398 | 0.213 | -1.866 | 0.062 | 0.186 |
| Vildgali*p*tin grou*p* | |  |  |  |  |  |
| TMAO | Baseline vs. M3 | 0.333 | 0.236 | -1.414 | 0.157 | 0.472 |
|  | Baseline vs. M6 | 0.750 | 0.236 | -3.182 | 0.001 | 0.004 |
|  | M3 vs. M6 | 0.417 | 0.236 | -1.768 | 0.077 | 0.231 |
| L-Carnitine | Baseline vs. 3M | -0.487 | 0.226 | -2.151 | 0.031 | 0.094 |
|  | Baseline vs. 6M | -0.897 | 0.226 | -3.963 | <0.001 | <0.001 |
|  | 3M vs. 6M | -0.410 | 0.226 | -1.812 | 0.070 | 0.210 |
| γ-Butyrobetaine | Baseline vs. 3M | -0.757 | 0.239 | -3.167 | 0.002 | 0.005 |
|  | Baseline vs. 6M | -0.871 | 0.239 | -3.645 | <0.001 | 0.001 |
|  | 3M vs. 6M | -0.114 | 0.239 | -0.478 | 0.633 | 1.000 |

Dunn's test was used for paired comparisons. *P*-values were corrected using the Bonferroni method.
