# Supplementary material for: Effect of acarbose and vildagliptin on plasma trimethylamine N-oxide levels in patients with type 2 diabetes mellitus: a 6-month, two-arm randomized controlled trial
Source: Front Endocrinol (Lausanne). 2025 May 6;16:1575087. doi: 10.3389/fendo.2025.1575087 (PMC12088947; doi:10.3389/fendo.2025.1575087)
Supplement: Supplementary file 3 [file Table3.docx]

Supplementary Material

**Supplementary Table 3-1 Association of changes in clinical metabolic characteristics with plasma levels of gut microbiota metabolites after 6 months of intervention**

| Acarbose grou*p* | | ΔTMAO | ΔL-Carnitine | ΔBetaine | ΔCholine | Δγ-Butyrobetaine | ΔWeight | ΔBMI | ΔWaist | ΔHip | ΔWHR | ΔHbA_1c_ | ΔFPG |
| --- | --- | --- | --- | --- | --- | --- | --- | --- | --- | --- | --- | --- | --- |
| ΔTMAO | r | 1 | 0.125 | 0.198 | 0.183 | 0.211 | 0.302 | 0.323* | 0.423** | 0.248 | 0.202 | 0.208 | 0.291 |
|  | *p* | . | 0.413 | 0.193 | 0.229 | 0.165 | 0.052 | 0.037 | 0.005 | 0.113 | 0.200 | 0.181 | 0.059 |
| ΔL-Carnitine | r | 0.125 | 1 | 0.092 | 0.181 | 0.644** | 0.188 | 0.216 | -0.225 | -0.09 | -0.116 | -0.139 | -0.138 |
|  | *p* | 0.413 | . | 0.547 | 0.234 | <0.001 | 0.233 | 0.17 | 0.151 | 0.57 | 0.465 | 0.372 | 0.376 |
| ΔBetaine | r | 0.198 | 0.092 | 1 | 0.657** | 0.199 | -0.059 | -0.128 | 0.013 | 0.136 | -0.108 | 0.314* | -0.120 |
|  | *p* | 0.193 | 0.547 | . | 0 | 0.189 | 0.712 | 0.42 | 0.937 | 0.389 | 0.495 | 0.041 | 0.445 |
| ΔCholine | r | 0.183 | 0.181 | 0.657** | 1 | 0.148 | -0.120 | -0.17 | -0.153 | -0.108 | 0.055 | 0.070 | -0.092 |
|  | *p* | 0.229 | 0.234 | <0.001 | . | 0.331 | 0.450 | 0.282 | 0.332 | 0.495 | 0.729 | 0.654 | 0.559 |
| Δγ-Butyrobetaine | r | 0.211 | 0.644** | 0.199 | 0.148 | 1 | 0.064 | 0.118 | -0.084 | -0.001 | -0.098 | -0.056 | -0.079 |
|  | *p* | 0.165 | <0.001 | 0.189 | 0.331 | . | 0.682 | 0.45 | 0.594 | 0.997 | 0.533 | 0.718 | 0.609 |
| ΔWeight | r | 0.302 | 0.188 | -0.059 | -0.120 | 0.064 | 1 | 0.987** | 0.325* | 0.238 | 0.044 | 0.208 | 0.188 |
|  | *p* | 0.052 | 0.233 | 0.712 | 0.450 | 0.682 | . | 0 | 0.026 | 0.107 | 0.770 | 0.160 | 0.205 |
| ΔBMI | r | 0.323* | 0.216 | -0.128 | -0.170 | 0.118 | 0.987** | 1 | 0.347* | 0.220 | 0.073 | 0.220 | 0.220 |
|  | *p* | 0.037 | 0.170 | 0.420 | 0.282 | 0.450 | 0 | . | 0.017 | 0.137 | 0.627 | 0.138 | 0.137 |
| ΔWaist | r | 0.423** | -0.225 | 0.013 | -0.153 | -0.084 | 0.325* | 0.347* | 1 | 0.455** | 0.475** | 0.435** | 0.530** |
|  | *p* | 0.005 | 0.151 | 0.937 | 0.332 | 0.594 | 0.026 | 0.017 | . | 0.001 | 0.001 | 0.002 | 0 |
| ΔHi*p* | r | 0.248 | -0.090 | 0.136 | -0.108 | -0.001 | 0.238 | 0.220 | 0.455** | 1 | -0.481** | 0.502** | 0.238 |
|  | *p* | 0.113 | 0.570 | 0.389 | 0.495 | 0.997 | 0.107 | 0.137 | 0.001 | . | 0.001 | 0 | 0.107 |
| ΔWHR | r | 0.202 | -0.116 | -0.108 | 0.055 | -0.098 | 0.044 | 0.073 | 0.475** | -.481** | 1 | -0.100 | 0.294* |
|  | *p* | 0.200 | 0.465 | 0.495 | 0.729 | 0.533 | 0.770 | 0.627 | 0.001 | 0.001 | . | 0.502 | 0.045 |
| ΔHbA_1c_ | r | 0.208 | -0.139 | 0.314* | 0.070 | -0.056 | 0.208 | 0.220 | 0.435** | .502** | -0.100 | 1 | 0.450** |
|  | *p* | 0.181 | 0.372 | 0.041 | 0.654 | 0.718 | 0.160 | 0.138 | 0.002 | 0 | 0.502 | . | 0.001 |
| ΔFPG | r | 0.291 | -0.138 | -0.120 | -0.092 | -0.079 | 0.188 | 0.220 | 0.530** | 0.238 | 0.294* | 0.450** | 1 |
|  | *p* | 0.059 | 0.376 | 0.445 | 0.559 | 0.609 | 0.205 | 0.137 | 0 | 0.107 | 0.045 | 0.001 | . |
| ΔPPG | r | 0.338* | -0.130 | -0.103 | 0.058 | -0.108 | 0.110 | 0.139 | 0.319* | -0.113 | 0.483** | 0.124 | 0.705** |
|  | *p* | 0.027 | 0.408 | 0.512 | 0.710 | 0.484 | 0.462 | 0.350 | 0.029 | 0.451 | 0.001 | 0.400 | 0 |
| ΔFins | r | 0.436** | 0.050 | -0.038 | 0.064 | -0.024 | 0.368* | 0.379** | 0.341* | 0.029 | 0.262 | 0.296* | 0.346* |
|  | *p* | 0.003 | 0.752 | 0.807 | 0.683 | 0.878 | 0.011 | 0.009 | 0.019 | 0.849 | 0.075 | 0.041 | 0.016 |
| ΔPins | r | 0.299 | -0.066 | -0.040 | 0.070 | -0.218 | 0.166 | 0.154 | 0.214 | -0.020 | 0.278 | 0.107 | 0.249 |
|  | *p* | 0.051 | 0.675 | 0.801 | 0.655 | 0.155 | 0.265 | 0.303 | 0.148 | 0.894 | 0.059 | 0.467 | 0.088 |
| ΔFc-peptide | r | 0.328* | 0.319* | 0.006 | 0.052 | 0.360* | 0.401** | 0.396** | 0.146 | 0.153 | 0.075 | 0.136 | 0.351* |
|  | *p* | 0.032 | 0.037 | 0.969 | 0.740 | 0.017 | 0.005 | 0.006 | 0.327 | 0.303 | 0.616 | 0.357 | 0.015 |
| ΔPc-peptide | r | 0.272 | 0.019 | -0.017 | 0.128 | 0.032 | -0.040 | -0.051 | -0.152 | 0.091 | -0.164 | -0.142 | 0.190 |
|  | *p* | 0.082 | 0.904 | 0.915 | 0.418 | 0.837 | 0.794 | 0.737 | 0.314 | 0.548 | 0.277 | 0.343 | 0.201 |
| ΔHOMA-IR | r | 0.484** | -0.017 | -0.100 | -0.031 | -0.020 | 0.323* | 0.341* | 0.393** | 0.046 | 0.301* | 0.342* | 0.600** |
|  | *p* | 0.001 | 0.915 | 0.523 | 0.846 | 0.897 | 0.027 | 0.019 | 0.006 | 0.760 | 0.040 | 0.017 | 0 |
| ΔHOMA-β | r | 0.214 | -0.019 | -0.077 | 0.061 | -0.047 | 0.285 | 0.278 | 0.175 | -0.069 | 0.184 | 0.008 | -0.119 |
|  | *p* | 0.169 | 0.901 | 0.622 | 0.696 | 0.764 | 0.052 | 0.059 | 0.240 | 0.644 | 0.217 | 0.957 | 0.422 |
| ΔGLP-1 | r | -0.041 | -0.136 | -0.096 | -0.063 | -0.136 | 0.257 | 0.234 | -0.031 | 0.091 | -0.101 | -0.111 | -0.033 |
|  | *p* | 0.789 | 0.371 | 0.531 | 0.682 | 0.368 | 0.082 | 0.113 | 0.838 | 0.544 | 0.501 | 0.451 | 0.822 |
| ΔCCK | r | 0.230 | -0.012 | 0.081 | -0.047 | 0.007 | 0.239 | 0.262 | 0.429** | 0.370* | 0.004 | 0.361* | 0.403** |
|  | *p* | 0.129 | 0.940 | 0.595 | 0.760 | 0.963 | 0.105 | 0.075 | 0.003 | 0.01 | 0.980 | 0.012 | 0.005 |
| ΔGhrelin | r | 0.047 | -0.024 | 0.524** | 0.285 | 0.150 | 0.044 | 0.009 | 0.155 | 0.302* | -0.134 | 0.258 | -0.273 |
|  | *p* | 0.757 | 0.876 | 0 | 0.058 | 0.320 | 0.771 | 0.952 | 0.297 | 0.039 | 0.370 | 0.076 | 0.060 |
| ΔPYY | r | 0.100 | 0.071 | -0.043 | 0.054 | 0.225 | 0.038 | 0.028 | -0.098 | 0.010 | -0.119 | -0.118 | 0.072 |
|  | *p* | 0.512 | 0.643 | 0.779 | 0.725 | 0.133 | 0.800 | 0.849 | 0.512 | 0.947 | 0.426 | 0.426 | 0.625 |
| Δleptin | r | 0.396** | -0.021 | 0.031 | -0.034 | -0.025 | 0.251 | 0.261 | 0.517** | 0.272 | 0.216 | 0.517** | 0.338* |
|  | *p* | 0.007 | 0.889 | 0.840 | 0.823 | 0.868 | 0.088 | 0.076 | 0 | 0.065 | 0.144 | 0 | 0.019 |
| Vildagliptin grou*p* | | |  |  |  |  |  |  |  |  |  |  |  |
| ΔTMAO | r | 1 | 0.102 | 0.145 | 0.143 | 0.182 | -0.022 | -0.009 | 0.027 | 0.223 | -0.213 | -0.156 | 0.087 |
|  | *p* | . | 0.541 | 0.387 | 0.399 | 0.275 | 0.898 | 0.957 | 0.874 | 0.191 | 0.213 | 0.358 | 0.609 |
| ΔCarnitine | r | 0.102 | 1 | -0.333* | 0.225 | 0.771** | 0.165 | 0.165 | 0.042 | -0.073 | 0.132 | -0.148 | 0.191 |
|  | *p* | 0.541 | . | 0.036 | 0.180 | 0 | 0.323 | 0.323 | 0.801 | 0.664 | 0.429 | 0.368 | 0.243 |
| ΔBetaine | r | 0.145 | -0.333* | 1 | 0.211 | -0.074 | -0.246 | -0.244 | 0.013 | -0.038 | 0.011 | 0.137 | 0.046 |
|  | *p* | 0.387 | 0.036 | . | 0.209 | 0.661 | 0.136 | 0.140 | 0.937 | 0.819 | 0.946 | 0.405 | 0.780 |
| ΔCholine | r | 0.143 | 0.225 | 0.211 | 1 | 0.152 | -0.010 | 0.003 | 0.012 | -0.198 | 0.255 | -0.039 | -0.013 |
|  | *p* | 0.399 | 0.180 | 0.209 | . | 0.368 | 0.952 | 0.985 | 0.944 | 0.253 | 0.139 | 0.821 | 0.939 |
| Δγ-Butyrobetaine | r | 0.182 | 0.771** | -0.074 | 0.152 | 1 | -0.038 | -0.035 | 0.001 | -0.101 | 0.073 | -0.156 | 0.292 |
|  | *p* | 0.275 | 0 | 0.661 | 0.368 | . | 0.827 | 0.842 | 0.998 | 0.558 | 0.674 | 0.357 | 0.079 |
| ΔWeight | r | -0.022 | 0.165 | -0.246 | -0.010 | -0.038 | 1 | 0.998** | 0.668** | 0.524** | 0.344* | 0.186 | 0.192 |
|  | *p* | 0.898 | 0.323 | 0.136 | 0.952 | 0.827 | . | 0 | 0 | 0 | 0.024 | 0.231 | 0.217 |
| ΔBMI | r | -0.009 | 0.165 | -0.244 | 0.003 | -0.035 | 0.998** | 1 | 0.670** | 0.520** | 0.350* | 0.172 | 0.191 |
|  | *p* | 0.957 | 0.323 | 0.140 | 0.985 | 0.842 | 0 | . | 0 | 0 | 0.021 | 0.269 | 0.219 |
| ΔWaist | r | 0.027 | 0.042 | 0.013 | 0.012 | 0.001 | 0.668** | 0.670** | 1 | 0.681** | 0.527** | 0.050 | 0.265 |
|  | *p* | 0.874 | 0.801 | 0.937 | 0.944 | 0.998 | 0 | 0 | . | 0 | 0 | 0.749 | 0.086 |
| ΔHip | r | 0.223 | -0.073 | -0.038 | -0.198 | -0.101 | 0.524** | 0.520** | 0.681** | 1 | -0.195 | 0.013 | .302* |
|  | *p* | 0.191 | 0.664 | 0.819 | 0.253 | 0.558 | 0 | 0 | 0 | . | 0.210 | 0.932 | 0.049 |
| ΔWHR | r | -0.213 | 0.132 | 0.011 | 0.255 | 0.073 | 0.344* | 0.350* | 0.527** | -0.195 | 1 | 0.143 | -0.026 |
|  | *p* | 0.213 | 0.429 | 0.946 | 0.139 | 0.674 | 0.024 | 0.021 | 0 | 0.210 | . | 0.360 | 0.866 |
| ΔHbA_1c_ | r | -0.156 | -0.148 | 0.137 | -0.039 | -0.156 | 0.186 | 0.172 | 0.050 | 0.013 | 0.143 | 1 | 0.431** |
|  | *p* | 0.358 | 0.368 | 0.405 | 0.821 | 0.357 | 0.231 | 0.269 | 0.749 | 0.932 | 0.360 | . | 0.003 |
| ΔFPG | r | 0.087 | 0.191 | 0.046 | -0.013 | 0.292 | 0.192 | 0.191 | 0.265 | 0.302* | -0.026 | 0.431** | 1 |
|  | *p* | 0.609 | 0.243 | 0.780 | 0.939 | 0.079 | 0.217 | 0.219 | 0.086 | 0.049 | 0.866 | 0.003 | . |
| ΔPPG | r | 0.004 | 0.099 | -0.087 | -0.044 | 0.107 | 0.115 | 0.108 | 0.086 | 0 | 0.064 | 0.386** | 0.655** |
|  | *p* | 0.981 | 0.550 | 0.597 | 0.800 | 0.529 | 0.464 | 0.491 | 0.583 | 0.998 | 0.682 | 0.010 | 0 |
| ΔFins | r | 0.264 | -0.061 | 0.285 | 0.018 | 0.114 | 0.024 | 0.036 | 0.482** | 0.364* | 0.231 | -0.040 | 0.156 |
|  | *p* | 0.114 | 0.713 | 0.079 | 0.916 | 0.501 | 0.876 | 0.818 | 0.001 | 0.016 | 0.136 | 0.799 | 0.311 |
| ΔPins | r | -0.005 | 0.010 | -0.009 | 0.043 | -0.049 | 0.188 | 0.201 | 0.285 | 0.099 | 0.207 | -0.178 | -0.081 |
|  | *p* | 0.977 | 0.950 | 0.955 | 0.805 | 0.776 | 0.227 | 0.197 | 0.064 | 0.529 | 0.182 | 0.249 | 0.602 |
| ΔFc-peptide | r | 0.228 | -0.081 | 0.060 | 0.056 | -0.087 | 0.225 | 0.243 | 0.327* | 0.260 | 0.150 | 0 | 0.135 |
|  | *p* | 0.174 | 0.624 | 0.716 | 0.746 | 0.607 | 0.147 | 0.116 | 0.032 | 0.092 | 0.337 | 0.999 | 0.383 |
| ΔPc-peptide | r | -0.165 | -0.007 | -0.114 | 0.125 | -0.164 | 0.209 | 0.224 | -0.046 | -0.212 | 0.121 | -0.074 | -0.085 |
|  | *p* | 0.328 | 0.965 | 0.488 | 0.467 | 0.333 | 0.179 | 0.149 | 0.768 | 0.172 | 0.441 | 0.631 | 0.584 |
| ΔHOMA-IR | r | 0.255 | 0.040 | 0.281 | 0.030 | 0.228 | 0.051 | 0.062 | 0.462** | 0.409** | 0.163 | 0.049 | 0.463** |
|  | *p* | 0.127 | 0.809 | 0.083 | 0.860 | 0.174 | 0.746 | 0.695 | 0.002 | 0.006 | 0.297 | 0.754 | 0.002 |
| ΔHOMA-β | r | 0.024 | -0.147 | 0.188 | -0.029 | -0.030 | -0.107 | -0.094 | 0.278 | 0.196 | 0.111 | -0.251 | -0.311* |
|  | *p* | 0.889 | 0.371 | 0.252 | 0.867 | 0.861 | 0.496 | 0.551 | 0.071 | 0.209 | 0.480 | 0.100 | 0.040 |
| ΔGLP-1 | r | -0.207 | -0.125 | 0.091 | 0.066 | -0.177 | 0.138 | 0.143 | 0.093 | -0.124 | 0.177 | -0.105 | -0.229 |
|  | *p* | 0.213 | 0.441 | 0.579 | 0.699 | 0.289 | 0.384 | 0.365 | 0.559 | 0.435 | 0.262 | 0.504 | 0.140 |
| ΔCCK | r | 0.132 | -0.253 | 0.170 | -0.218 | -0.104 | -0.104 | -0.109 | -0.027 | -0.034 | 0.004 | 0.222 | 0.110 |
|  | *p* | 0.430 | 0.115 | 0.295 | 0.195 | 0.535 | 0.514 | 0.491 | 0.867 | 0.833 | 0.979 | 0.152 | 0.483 |
| ΔGhrelin | r | 0.101 | -0.090 | 0.272 | 0.004 | 0.188 | -0.311* | -0.307* | -0.239 | -0.166 | -0.182 | 0.053 | 0.299 |
|  | *p* | 0.547 | 0.579 | 0.090 | 0.980 | 0.259 | 0.045 | 0.048 | 0.128 | 0.294 | 0.248 | 0.734 | 0.051 |
| ΔPYY | r | 0.188 | -0.239 | 0.244 | -0.101 | -0.120 | -0.238 | -0.218 | -0.038 | -0.138 | 0.013 | -0.088 | -0.004 |
|  | *p* | 0.259 | 0.137 | 0.129 | 0.553 | 0.473 | 0.129 | 0.166 | 0.809 | 0.383 | 0.935 | 0.573 | 0.981 |
| Δleptin | r | 0.032 | -0.036 | 0.042 | -0.111 | 0.109 | 0.179 | 0.191 | 0.305* | 0.147 | 0.298 | 0.055 | 0.019 |
|  | *p* | 0.846 | 0.824 | 0.798 | 0.512 | 0.514 | 0.258 | 0.225 | 0.050 | 0.352 | 0.056 | 0.725 | 0.904 |

Spearman bivariate correlation analysis was used to analyse the changes (
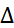
) of each variable to its baseline with consistent responses to 6-month treatment with acarbose or vildagliptin treatment. r:Spearman rank correlation coefficients (Spearman's rho) ; **p*<0.05, ***p*<0.01, ****p*<0.001.

**Supplementary Table 3-2 Association of changes in clinical metabolic characteristics with plasma levels of gut microbiota metabolites after 6 months of intervention**

| Acarbose group | | ΔPPG | ΔFins | ΔPins | ΔFc-peptide | ΔPc-peptide | ΔHOMA-IR | ΔHOMA-β | ΔGLP-1 | ΔCCK | ΔGhrelin | ΔPYY | Δleptin |
| --- | --- | --- | --- | --- | --- | --- | --- | --- | --- | --- | --- | --- | --- |
| ΔTMAO | r | 0.338* | 0.436** | 0.299 | 0.328* | 0.272 | 0.484** | 0.214 | -0.041 | 0.230 | 0.047 | 0.100 | 0.396** |
|  | *p* | 0.027 | 0.003 | 0.051 | 0.032 | 0.082 | 0.001 | 0.169 | 0.789 | 0.129 | 0.757 | 0.512 | 0.007 |
| ΔCarnitine | r | -0.130 | 0.050 | -0.066 | 0.319* | 0.019 | -0.017 | -0.019 | -0.136 | -0.012 | -0.024 | 0.071 | -0.021 |
|  | *p* | 0.408 | 0.752 | 0.675 | 0.037 | 0.904 | 0.915 | 0.901 | 0.371 | 0.940 | 0.876 | 0.643 | 0.889 |
| ΔBetaine | r | -0.103 | -0.038 | -0.040 | 0.006 | -0.017 | -0.100 | -0.077 | -0.096 | 0.081 | 0.524** | -0.043 | 0.031 |
|  | *p* | 0.512 | 0.807 | 0.801 | 0.969 | 0.915 | 0.523 | 0.622 | 0.531 | 0.595 | <0.001 | 0.779 | 0.840 |
| ΔCholine | r | 0.058 | 0.064 | 0.070 | 0.052 | 0.128 | -0.031 | 0.061 | -0.063 | -0.047 | 0.285 | 0.054 | -0.034 |
|  | *p* | 0.710 | 0.683 | 0.655 | 0.740 | 0.418 | 0.846 | 0.696 | 0.682 | 0.760 | 0.058 | 0.725 | 0.823 |
| ΔButyrobetaine | r | -0.108 | -0.024 | -0.218 | 0.360* | 0.032 | -0.020 | -0.047 | -0.136 | 0.007 | 0.150 | 0.225 | -0.025 |
|  | *p* | 0.484 | 0.878 | 0.155 | 0.017 | 0.837 | 0.897 | 0.764 | 0.368 | 0.963 | 0.320 | 0.133 | 0.868 |
| ΔWeight | r | 0.110 | 0.368* | 0.166 | 0.401** | -0.040 | 0.323* | 0.285 | 0.257 | 0.239 | 0.044 | 0.038 | 0.251 |
|  | *p* | 0.462 | 0.011 | 0.265 | 0.005 | 0.794 | 0.027 | 0.052 | 0.082 | 0.105 | 0.771 | 0.800 | 0.088 |
| ΔBMI | r | 0.139 | 0.379** | 0.154 | 0.396** | -0.051 | 0.341* | 0.278 | 0.234 | 0.262 | 0.009 | 0.028 | 0.261 |
|  | *p* | 0.350 | 0.009 | 0.303 | 0.006 | 0.737 | 0.019 | 0.059 | 0.113 | 0.075 | 0.952 | 0.849 | 0.076 |
| ΔWaist | r | 0.319* | .341* | 0.214 | 0.146 | -0.152 | 0.393** | 0.175 | -0.031 | 0.429** | 0.155 | -0.098 | 0.517** |
|  | *p* | 0.029 | 0.019 | 0.148 | 0.327 | 0.314 | 0.006 | 0.24 | 0.838 | 0.003 | 0.297 | 0.512 | <0.001 |
| ΔHi*p* | r | -0.113 | 0.029 | -0.020 | 0.153 | 0.091 | 0.046 | -0.069 | 0.091 | 0.370* | 0.302* | 0.010 | 0.272 |
|  | *p* | 0.451 | 0.849 | 0.894 | 0.303 | 0.548 | 0.760 | 0.644 | 0.544 | 0.010 | 0.039 | 0.947 | 0.065 |
| ΔWHR | r | 0.483** | 0.262 | 0.278 | 0.075 | -0.164 | 0.301* | 0.184 | -0.101 | 0.004 | -0.134 | -0.119 | 0.216 |
|  | *p* | 0.001 | 0.075 | 0.059 | 0.616 | 0.277 | 0.040 | 0.217 | 0.501 | 0.980 | 0.370 | 0.426 | 0.144 |
| ΔHbA1c | r | 0.124 | 0.296* | 0.107 | 0.136 | -0.142 | 0.342* | 0.008 | -0.111 | 0.361* | 0.258 | -0.118 | 0.517** |
|  | *p* | 0.400 | 0.041 | 0.467 | 0.357 | 0.343 | 0.017 | 0.957 | 0.451 | 0.012 | 0.076 | 0.426 | 0 |
| ΔF*p*G | r | 0.705** | 0.346* | 0.249 | 0.351* | 0.190 | 0.600** | -0.119 | -0.033 | 0.403** | -0.273 | 0.072 | 0.338* |
|  | *p* | 0 | 0.016 | 0.088 | 0.015 | 0.201 | 0 | 0.422 | 0.822 | 0.005 | 0.060 | 0.625 | 0.019 |
| Δ*pp*G | r | 1 | 0.185 | 0.335* | 0.217 | 0.265 | 0.403** | -0.114 | 0.065 | 0.222 | -0.361* | -0.054 | 0.236 |
|  | *p* | . | 0.208 | 0.020 | 0.139 | 0.071 | 0.004 | 0.441 | 0.660 | 0.129 | 0.012 | 0.717 | 0.106 |
| ΔFins | r | 0.185 | 1 | 0.345* | .439** | -0.055 | 0.932** | 0.789** | 0.015 | 0.288* | 0.021 | 0.090 | 0.392** |
|  | *p* | 0.208 | . | 0.016 | 0.002 | 0.714 | 0 | 0 | 0.917 | 0.047 | 0.886 | 0.541 | 0.006 |
| Δ*p*ins | r | 0.335* | 0.345* | 1 | 0.079 | 0.518** | 0.346* | 0.261 | 0.070 | 0.314* | -0.137 | -0.079 | 0.296* |
|  | *p* | 0.020 | 0.016 | . | 0.592 | 0 | 0.016 | 0.073 | 0.636 | 0.029 | 0.354 | 0.595 | 0.041 |
| ΔFc-*p*e*p*tide | r | 0.217 | 0.439** | 0.079 | 1 | 0.369* | 0.462** | 0.269 | 0.140 | 0.085 | -0.135 | 0.224 | 0.141 |
|  | *p* | 0.139 | 0.002 | 0.592 | . | 0.011 | 0.001 | 0.064 | 0.341 | 0.563 | 0.361 | 0.126 | 0.338 |
| Δ*p*c-*p*e*p*tide | r | 0.265 | -0.055 | 0.518** | 0.369* | 1 | 0.001 | -0.125 | 0.212 | 0.212 | -0.179 | 0.253 | -0.008 |
|  | *p* | 0.071 | 0.714 | 0 | 0.011 | . | 0.996 | 0.404 | 0.153 | 0.153 | 0.228 | 0.087 | 0.958 |
| ΔHOMA-IR | r | 0.403** | 0.932** | 0.346* | .462** | 0.001 | 1 | .583** | -0.004 | 0.317* | -0.093 | 0.111 | 0.428** |
|  | *p* | 0.004 | 0 | 0.016 | 0.001 | 0.996 | . | 0 | 0.977 | 0.028 | 0.527 | 0.454 | 0.002 |
| ΔHOMA-β | r | -0.114 | 0.789** | 0.261 | 0.269 | -0.125 | 0.583** | 1 | 0.033 | 0.137 | 0.092 | 0.026 | 0.155 |
|  | *p* | 0.441 | 0 | 0.073 | 0.064 | 0.404 | 0 | . | 0.823 | 0.352 | 0.533 | 0.860 | 0.294 |
| ΔGL*p*-1 | r | 0.065 | 0.015 | 0.070 | 0.140 | 0.212 | -0.004 | 0.033 | 1 | 0.226 | 0.051 | 0.088 | -0.080 |
|  | *p* | 0.660 | 0.917 | 0.636 | 0.341 | 0.153 | 0.977 | 0.823 | . | 0.115 | 0.725 | 0.545 | 0.580 |
| ΔCCK | r | 0.222 | 0.288* | 0.314* | 0.085 | 0.212 | 0.317* | 0.137 | 0.226 | 1 | 0.061 | 0.071 | 0.401** |
|  | *p* | 0.129 | 0.047 | 0.029 | 0.563 | 0.153 | 0.028 | 0.352 | 0.115 | . | 0.675 | 0.624 | 0.004 |
| ΔGhrelin | r | -0.361* | 0.021 | -0.137 | -0.135 | -0.179 | -0.093 | 0.092 | 0.051 | 0.061 | 1 | 0.004 | 0.062 |
|  | *p* | 0.012 | 0.886 | 0.354 | 0.361 | 0.228 | 0.527 | 0.533 | 0.725 | 0.675 | . | 0.981 | 0.671 |
| Δ*p*YY | r | -0.054 | 0.090 | -0.079 | 0.224 | 0.253 | 0.111 | 0.026 | 0.088 | 0.071 | 0.004 | 1 | -0.083 |
|  | *p* | 0.717 | 0.541 | 0.595 | 0.126 | 0.087 | 0.454 | 0.86 | 0.545 | 0.624 | 0.981 | . | 0.569 |
| Δle*p*tin | r | 0.236 | 0.392** | 0.296* | 0.141 | -0.008 | 0.428** | 0.155 | -0.080 | 0.401** | 0.062 | -0.083 | 1 |
|  | *p* | 0.106 | 0.006 | 0.041 | 0.338 | 0.958 | 0.002 | 0.294 | 0.580 | 0.004 | 0.671 | 0.569 | . |
| Vildagli*p*tin grou*p* | | | | | | | | | | | | | |
| ΔTMAO | r | 0.004 | 0.264 | -0.005 | 0.228 | -0.165 | 0.255 | 0.024 | -0.207 | 0.132 | 0.101 | 0.188 | 0.032 |
|  | *p* | 0.981 | 0.114 | 0.977 | 0.174 | 0.328 | 0.127 | 0.889 | 0.213 | 0.430 | 0.547 | 0.259 | 0.846 |
| ΔCarnitine | r | 0.099 | -0.061 | 0.010 | -0.081 | -0.007 | 0.040 | -0.147 | -0.125 | -0.253 | -0.090 | -0.239 | -0.036 |
|  | *p* | 0.550 | 0.713 | 0.950 | 0.624 | 0.965 | 0.809 | 0.371 | 0.441 | 0.115 | 0.579 | 0.137 | 0.824 |
| ΔBetaine | r | -0.087 | 0.285 | -0.009 | 0.060 | -0.114 | 0.281 | 0.188 | 0.091 | 0.170 | 0.272 | 0.244 | 0.042 |
|  | *p* | 0.597 | 0.079 | 0.955 | 0.716 | 0.488 | 0.083 | 0.252 | 0.579 | 0.295 | 0.090 | 0.129 | 0.798 |
| ΔCholine | r | -0.044 | 0.018 | 0.043 | 0.056 | 0.125 | 0.030 | -0.029 | 0.066 | -0.218 | 0.004 | -0.101 | -0.111 |
|  | *p* | 0.800 | 0.916 | 0.805 | 0.746 | 0.467 | 0.860 | 0.867 | 0.699 | 0.195 | 0.980 | 0.553 | 0.512 |
| ΔButyrobetaine | r | 0.107 | 0.114 | -0.049 | -0.087 | -0.164 | 0.228 | -0.030 | -0.177 | -0.104 | 0.188 | -0.120 | 0.109 |
|  | *p* | 0.529 | 0.501 | 0.776 | 0.607 | 0.333 | 0.174 | 0.861 | 0.289 | 0.535 | 0.259 | 0.473 | 0.514 |
| ΔWeight | r | 0.115 | 0.024 | 0.188 | 0.225 | 0.209 | 0.051 | -0.107 | 0.138 | -0.104 | -0.311* | -0.238 | 0.179 |
|  | *p* | 0.464 | 0.876 | 0.227 | 0.147 | 0.179 | 0.746 | 0.496 | 0.384 | 0.514 | 0.045 | 0.129 | 0.258 |
| ΔBMI | r | 0.108 | 0.036 | 0.201 | 0.243 | 0.224 | 0.062 | -0.094 | 0.143 | -0.109 | -0.307* | -0.218 | 0.191 |
|  | *p* | 0.491 | 0.818 | 0.197 | 0.116 | 0.149 | 0.695 | 0.551 | 0.365 | 0.491 | 0.048 | 0.166 | 0.225 |
| ΔWaist | r | 0.086 | 0.482** | 0.285 | 0.327* | -0.046 | 0.462** | 0.278 | 0.093 | -0.027 | -0.239 | -0.038 | 0.305* |
|  | *p* | 0.583 | 0.001 | 0.064 | 0.032 | 0.768 | 0.002 | 0.071 | 0.559 | 0.867 | 0.128 | 0.809 | 0.050 |
| ΔHi*p* | r | 0 | 0.364* | 0.099 | 0.260 | -0.212 | 0.409** | 0.196 | -0.124 | -0.034 | -0.166 | -0.138 | 0.147 |
|  | *p* | 0.998 | 0.016 | 0.529 | 0.092 | 0.172 | 0.006 | 0.209 | 0.435 | 0.833 | 0.294 | 0.383 | 0.352 |
| ΔWHR | r | 0.064 | 0.231 | 0.207 | 0.150 | 0.121 | 0.163 | 0.111 | 0.177 | 0.004 | -0.182 | 0.013 | 0.298 |
|  | *p* | 0.682 | 0.136 | 0.182 | 0.337 | 0.441 | 0.297 | 0.480 | 0.262 | 0.979 | 0.248 | 0.935 | 0.056 |
| ΔHbA1c | r | 0.386** | -0.040 | -0.178 | 0 | -0.074 | 0.049 | -0.251 | -0.105 | 0.222 | 0.053 | -0.088 | 0.055 |
|  | *p* | 0.010 | 0.799 | 0.249 | 0.999 | 0.631 | 0.754 | 0.100 | 0.504 | 0.152 | 0.734 | 0.573 | 0.725 |
| ΔF*p*G | r | 0.655** | 0.156 | -0.081 | 0.135 | -0.085 | 0.463** | -0.311* | -0.229 | 0.110 | 0.299 | -0.004 | 0.019 |
|  | *p* | 0 | 0.311 | 0.602 | 0.383 | 0.584 | 0.002 | 0.040 | 0.140 | 0.483 | 0.051 | 0.981 | 0.904 |
| Δ*pp*G | r | 1 | 0.095 | 0.259 | 0.297 | 0.192 | 0.277 | -0.206 | 0.111 | 0.190 | 0.265 | -0.032 | 0.043 |
|  | *p* | . | 0.537 | 0.089 | 0.05 | 0.212 | 0.068 | 0.181 | 0.480 | 0.223 | 0.085 | 0.837 | 0.784 |
| ΔFins | r | 0.095 | 1 | 0.373* | 0.665** | -0.099 | 0.910** | 0.777** | 0.045 | 0.093 | -0.112 | 0.287 | 0.424** |
|  | *p* | 0.537 | . | 0.013 | 0 | 0.522 | 0 | 0 | 0.775 | 0.555 | 0.473 | 0.062 | 0.005 |
| Δ*p*ins | r | 0.259 | 0.373* | 1 | 0.419** | 0.519** | 0.269 | 0.450** | 0.369* | 0.056 | 0.038 | -0.009 | 0.267 |
|  | *p* | 0.089 | 0.013 | . | 0.005 | 0 | 0.078 | 0.002 | 0.015 | 0.721 | 0.809 | 0.956 | 0.083 |
| ΔFc-*p*e*p*tide | r | 0.297 | 0.665** | 0.419** | 1 | 0.326* | 0.592** | .478** | 0.279 | -0.008 | -0.130 | 0.288 | 0.299 |
|  | *p* | 0.050 | 0 | 0.005 | . | 0.031 | 0 | 0.001 | 0.070 | 0.958 | 0.407 | 0.061 | 0.051 |
| Δ*p*c-*p*e*p*tide | r | 0.192 | -0.099 | 0.519** | 0.326* | 1 | -0.168 | -0.038 | 0.269 | 0.043 | -0.001 | 0.235 | -0.031 |
|  | *p* | 0.212 | 0.522 | 0 | 0.031 | . | 0.275 | 0.809 | 0.081 | 0.786 | 0.994 | 0.129 | 0.843 |
| ΔHOMA-IR | r | 0.277 | 0.910** | 0.269 | 0.592** | -0.168 | 1 | 0.528** | -0.122 | 0.051 | 0.007 | 0.243 | 0.431** |
|  | *p* | 0.068 | 0 | 0.078 | 0 | 0.275 | . | 0 | 0.435 | 0.744 | 0.963 | 0.117 | 0.004 |
| ΔHOMA-β | r | -0.206 | 0.777** | 0.450** | 0.478** | -0.038 | 0.528** | 1 | 0.267 | 0.021 | -0.143 | 0.258 | 0.307* |
|  | *p* | 0.181 | 0 | 0.002 | 0.001 | 0.809 | 0 | . | 0.083 | 0.891 | 0.361 | 0.095 | 0.046 |
| ΔGL*p*-1 | r | 0.111 | 0.045 | 0.369* | 0.279 | 0.269 | -0.122 | 0.267 | 1 | 0.105 | -0.034 | -0.075 | 0.045 |
|  | *p* | 0.480 | 0.775 | 0.015 | 0.070 | 0.081 | 0.435 | 0.083 | . | 0.499 | 0.827 | 0.630 | 0.771 |
| ΔCCK | r | 0.190 | 0.093 | 0.056 | -0.008 | 0.043 | 0.051 | 0.021 | 0.105 | 1 | 0.185 | 0.106 | -0.091 |
|  | *p* | 0.223 | 0.555 | 0.721 | 0.958 | 0.786 | 0.744 | 0.891 | 0.499 | . | 0.229 | 0.492 | 0.555 |
| ΔGhrelin | r | 0.265 | -0.112 | 0.038 | -0.130 | -0.001 | 0.007 | -0.143 | -0.034 | 0.185 | 1 | -0.112 | -0.072 |
|  | *p* | 0.085 | 0.473 | 0.809 | 0.407 | 0.994 | 0.963 | 0.361 | 0.827 | 0.229 | . | 0.470 | 0.642 |
| Δ*p*YY | r | -0.032 | 0.287 | -0.009 | 0.288 | 0.235 | 0.243 | 0.258 | -0.075 | 0.106 | -0.112 | 1 | 0.139 |
|  | *p* | 0.837 | 0.062 | 0.956 | 0.061 | 0.129 | 0.117 | 0.095 | 0.630 | 0.492 | 0.470 | . | 0.367 |
| Δle*p*tin | r | 0.043 | 0.424** | 0.267 | 0.299 | -0.031 | 0.431** | 0.307* | 0.045 | -0.091 | -0.072 | 0.139 | 1 |
|  | *p* | 0.784 | 0.005 | 0.083 | 0.051 | 0.843 | 0.004 | 0.046 | 0.771 | 0.555 | 0.642 | 0.367 | . |

Spearman bivariate correlation analysis was used to analyse the changes (
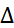
) of each variable to its baseline with consistent responses to 6-month treatment with acarbose or vildagliptin treatment. r:Spearman rank correlation coefficients (Spearman's rho) ; **p*<0.05, ***p*<0.01, ****p*<0.001.
